# Notes and News

**Published:** 1888-08-11

**Authors:** 


### NOTES AND NEWS.

COLLECTED AND COMMUNICATED.

THE Earl of Aberdeen has accepted the position of president to the Morley House Seaside Convalescent Home, established in connexion with the Hospital Saturday Fund.

ON July 23rd, M. Waddington, the French Ambassador, performed the ceremony of laying the first stone of the new French Hospital which is to be erected in Shaftesbury Avenue.

THE Clackmannan County Hospital has a ladies' committee, which meets every Friday to audit the accounts of the housekeeping department, and generally exercises, with the aid of the matron, a surveillance in household matters.

THE Aberdeen Infirmary and Lunatic Asylum Corporation have agreed to purchase the mansion-house of Glack, with 283 acres of land in the parish of Daviot, at a cost of £11,000, to be used as a branch asylum for lunatic patients.

AT the annual meeting of the Hereford Dispensary it was stated that the provident branch was increasing in membership, the amount subscribed in weekly pence being £30 more this year than last.

THE daily average of patients at the Huddersfield Infirmary during the year ending June 30th was 75. The total number treated in the wards was 921, and the out-patients numbered 3,977, besides 964 cases which have been treated at their own homes by the hospital doctors and nurses.

AN International Medical Congress on Tubercular Diseases held its opening sitting in Paris, at the Sorbonne, on the 26th of July. Its object, as set forth by Professor Verneuil, is to ascertain as accurately as possible, from comparisons between man and the domestic animals, the origin, the conditions, and the course of tuberculosis. This research it may be hoped will lead to the discovery of the most rational and the best methods of arresting and even curing that most fatal disease, which causes more deaths than all the other contagious diseases put together. The sittings of the Congress are full of promise. More than two hundred medical men, French and foreign, have read papers.

THE *Alliance*, a floating hospital for infectious diseases sank in the Tyne on July 26th. Fortunately no one was on board her.

LATE on Saturday night, the 21st ult., through the kind thoughtfulness of Mrs. Bernard Beere, the patients of the Great Ormond Street Children's Hospital received some of the beautiful bouquets and baskets of flowers which had been presented to her on her benefit at the Opera Comique, which took place on that date.

TWELVE students of the London School of Medicine for Women presented themselves for the first and second examinations of the Royal Colleges of Physicians and Surgeons of Edinburgh, and all passed. Miss Waterston, M.D., formerly a student of the London School of Medicine for Women, has just passed the examinations of the Psychological Society for a certificate in mental diseases. Miss Waterston is the first lady who has presented herself for this examination.

AT a meeting of the board of management of the Manchester Royal Infirmary, Mr. Heywood, who presided, said he had an hour previously returned from the Monsall Hospital, and had received the latest particulars of an alarming outbreak of small-pox at St. Joseph's Industrial School. At that time there were at the hospital three sisters, five attendants, and fifty-eight children under treatment, thirteen of the children being seriously ill.

AT the annual meeting of the Manchester Royal Infirmary, Mr. E. S. Heywood, commenting on the fact that the number of patients treated at the infirmary and the institutions affiliated to it during the last year had been 41,373, while five years ago they were little more than half that number, said that these figures pointed to the fact that "a large proportion of the working-class population was either unwilling or unable to pay for medical attendance." Whichever hypothesis be the true one, it points to a very serious social condition, which cannot be too soon remedied.

LADY SMYTH's home, near Bristol, for females broken down in health, and requiring rest, quiet, and fresh air—so necessary to the multitude of toilers in this work-a-day world—is a welcome addition to those beneficent establishments which are becoming more and more recognised by physicians as a means of restoring those whose strength has been shattered by labour and care. The house, which is on the hill at Long Ashton, is beautifully situated, being 300 feet above the level of the sea and 250 feet above the Ashton Valley. It commands a most magnificent and extensive view—to Dundry in front, to Lansdown on the left, and a considerable distance on the right. The home is almost immediately underneath a clump of fir trees—a landmark for many miles—by which, together with the high green plateau behind, it is sheltered from the north and north-west winds.

The following vacancies are announced this week, the amount of salary in each case being given after the name of the institution :—

**Resident Medical Officers.**

Belgrave Hospital for Children. Resident House Surgeon and Assistant Secretary.—Salary at the rate of £30 per annum, with board, etc.
East Suffolk and Ipswich Hospital. Assistant House Surgeon.—£20 per annum, with board, etc.
General Hospital, Nottingham. Senior Resident Medical Officer.—£120 per annum, with increase, board, etc.
Parish of St. Matthew, Bethnal Green. Assistant Medical Officer.—£100 per annum, with board, etc.
Swansea Hospital. Resident Medical Officer.—£100 per annum, with board, etc.

**Non-resident Medical Officers.**

Carrick-on-Suir Union. Medical Officer.
Hulme Dispensary, Manchester. Honorary Surgeon.
Italian Hospital, Queen Square, Bloomsbury. Assistant Medical Officer.
Rathdrum Union. Medical Officer.—£110 per annum, and fees.
University College, Bristol. Medical Tutor.—£125 per annum.

An interesting series of articles on "The Genesis, Growth, and Work of the Blackburn and East Lancashire Infirmary" has just begun to appear in the *Blackburn Standard*.

Saturday, October 8th, has been fixed for the Hospital Saturday collection at Colchester. This is rather a late date; but in view of the wet summer, and the hopes now beginning to be cherished of a fine autumn, it is thought that the collection may be more successful then than it would be earlier.

The Government of Bombay has recommended that Sir Dinshaw Maneckjee Petit's munificent gift of a lakh and a-quarter of rupees should be devoted to the building of a hospital for women and children in connexion with the Jamsetjee Jejeebhoy Obstetric Hospital. Sir Dinshaw Petit has assented to the plan.

The contributors to the Jubilee offering of womenkind to the Queen came from every part of the Empire, in proportion to the population, as may be seen from the following figures: England, 2,206,122; Scotland, 415,165; Ireland, 158,620; Wales, 172,948; Guernsey, Alderney and Sark, 8,883; Jersey, 4,703; Isle of Man, 3,290; Y. W. C. Association, 16,666; foreign, per Lady Paget (£1,035), and various, 5,859; Ceylon, 100,000; Burmah, 70,000. Grand total, 3,162,256.

The work of the executive of the Women's Jubilee Offering is now drawing to a close. The total contributions from three and a-quarter millions of the "daughters of the Empire," amounting to £84,116 net, have so far been dealt with as follows: £70,000 in cash has been transferred to the trustees nominated by the Queen to act in the matter of the Nursing Fund—viz., the Duke of Westminster, K.G., Sir Rutherford Alcock, K.C.B., and Sir James Paget, Bart. The model of the statue of the Prince Consort, to be erected in Windsor Park will soon be ready for casting at the foundry, and her Majesty has received from the hands of the Duchess of Buccleuch, the president of the Committee of Selection, the pearl and diamond necklace and earrings purchased out of the surplus after providing for the Nursing Fund and the statue. The personal present to the Queen was manufactured by Messrs. Carrington and Co., of Regent Street, and consists of pearls of unusual size and colour, set in diamonds and in a trefoil pattern, enclosed in a cabinet bearing the inscription: "To Victoria, Queen and Empress; a token of love and loyalty, from the daughters of her Empire, in remembrance of her Jubilee, June 21, 1887." The larger sections of the necklace take to pieces, and can be worn as pins or brooches. With the sanction of her Majesty, the ornaments were, by permission of the Duchess of Buccleuch, shown to the members of the general committee on Thursday and Friday, at Montagu House. It is hoped that the sanction of the Queen will be obtained for the more public exhibition of the ornaments during the autumn.

It is proposed to erect a convalescent home for members of friendly societies on the north-eastern coast. The plan originated with Dr. W. Robinson, of Stanhope, and has met with the hearty approval of most of the gentlemen interested in friendly societies in the north-eastern counties.

As the result of a canvas, undertaken with the object of ascertaining the popular feeling of the East-end of London with regard to the Sunday opening of the People's Palace, it was found that 55,717 were in favour of it, whilst only 20,000 signed against it. The Palace is opened on Sundays.

It is stated that of the 600 beds at the Glasgow Royal Infirmary, 400 are for surgical cases; and that more surgical operations of an important character are performed at the infirmary than at any other hospital in the kingdom. No fewer than three operation theatres are in constant use.

The committee of the Royal Albert Hospital, Devonport, have resolved to establish a nursing institute in connexion with the hospital. At present, the measure is merely tentative, but if the demands for the services of trained nurses for private work continue to be as numerous as they have lately been, the institute will become a permanently established fact.

A hospital has been erected at the works at Salford, where the making of the long-desired Manchester Ship Canal is now proceeding. It is only a wooden building, as there will be no permanent need for it, but it is fitted with all the necessary drugs and appliances; and Dr. Cran, the medical man appointed to the charge of it, also attends the families of the workmen at their homes. There is also a properly-trained ambulance class in connexion with the works.

The "Byram" Convalescent Home for Ladies, Wordsworth Road, Worthing, combines the double advantage of being a place where ladies can go for change of air on moderate terms, and where convalescents are nursed back to complete health by competent trained nurses. A few cases of non-infectious illness are also taken at an advance on the charge for convalescents, which is only 15*s.* a-week for adults, and 12*s.* 6*d.* a-week for children. As the institution is moderate in size, a more home-like feeling is obtained than can easily be managed in a larger place.

The Board of Superintendence of the Dublin Hospitals receiving aid from Parliament have submitted their thirtieth report to the Lord Lieutenant, and it appears to be an eminently satisfactory document. In all of the institutions inspected the general management was found satisfactory. The number admitted during the year was 9,875, making the total under treatment 10,635, of whom 9,376 left either cured or relieved, or were discharged for other causes, and 512 died. On March 31st this year 740 patients remained in the nine hospitals inspected, including those of the House of Industry. The mortality was 5·39 per cent. of those treated to a termination, and the total average daily number of beds occupied throughout the year was 744·57.

A man of gentlemanly appearance, about fifty years of age, committed suicide a few days ago by cutting his throat in a bath at Marylebone. A letter was found on the seat of the bath, the contents of which were as follows: "I have been most cruelly treated and grossly deceived in my relations with Annie ——. She has broken my heart, so that life is no longer endurable. May she endure a tithe of the misery she has caused me. Oh, how I loved her! It is maddening. I was not aware until lately that she is a wife. She is a thorough demoness. She has ruined me in every way.—A. C. D." The jury returned a verdict of "Suicide while of unsound mind."

